# Characteristics of hindfoot morphology and ankle range of motion in young women with hallux valgus

**DOI:** 10.1186/s13047-023-00666-4

**Published:** 2023-09-25

**Authors:** Mieko Yokozuka, Kanako Okazaki

**Affiliations:** Department of Physical Therapy, Fukushima Medical University School of Health Sciences, 10-6 Sakae-machi, Fukushima City, Fukushima 960-8516 Japan

**Keywords:** Hallux valgus, Morphology, Posture, Pronation, Ankle

## Abstract

**Background:**

Hallux valgus occurs more frequently in women as they age; therefore, it is beneficial to prevent hallux valgus in younger women. The purpose of this study was to clarify the characteristics of hindfoot morphology and the range of motion of the ankle joint with hallux valgus in young women.

**Methods:**

The participants were 140 young women (mean age 18.8 ± 0.6 years). A three-dimensional footprint automatic measurement apparatus was used to measure the hallux valgus angle in the standing position and the arch-height ratio and heel-floor angle (HFA) in the standing and chair-sitting positions. The amount of change in foot morphology owing to differences in posture was calculated. The range of motion of the ankle joint dorsiflexion, plantarflexion, inversion, and eversion was measured. Participants were classified into two groups according to the presence of hallux valgus. Statistical analysis was used to compare hindfoot morphology and range of motion between the two groups, and the correlation between foot morphology and range of motion was investigated depending on the presence of hallux valgus.

**Results:**

With hallux valgus, the HFA tilted inwards (*p* = 0.010), and the change in the arch-height ratio due to the difference in posture was large (*p* = 0.021). There was no difference in the range of motion of the ankle joints with or without hallux valgus. In women with hallux valgus, the amount of change in arch height and HFA was correlated with the range of motion of eversion (*r* = 0.391, *p* = 0.027; *r* = -0.362, *p* = 0.042).

**Conclusions:**

With hallux valgus, the hindfoot pronated, and the arch height decreased from sitting to standing. Furthermore, the amount of change in the hindfoot and midfoot due to posture was related to the range of motion of eversion.

## Background

The prevalence of hallux valgus, a foot deformity, is higher in women than in men [1]. Based on measurements using a foot printer, hallux valgus was found in 29.7–41.5% of Japanese female university students [2, 3]. Regarding hallux valgus and motor function in young women, feet with hallux valgus were shown to have significantly reduced toe flexor strength, and a correlation was found between toe flexor strength and maximum step length [3]. Although a causal relationship has not been established, it is important to prevent hallux valgus from an early stage because feet with hallux valgus show decreased motor function.

It has been reported that the pronation of the metatarsals is characteristic of feet with hallux valgus [4, 5]. Regarding the morphology of the hallux valgus and midfoot, the foot pressure distribution during walking on the hallux valgus showed that the foot pressure on the lateral side of the midfoot significantly decreased [3], which is associated with midfoot pronation and decreased modified arch index [6, 7]. With regard to the hindfoot of hallux valgus, a correlation has been observed between the hallux valgus angle and inward inclination angle of the calcaneus in older women [8]. In medial knee osteoarthritis, a relationship between the hallux valgus angle and medial tilt of the calcaneus has been reported [9–11]. Conversely, it has also been reported that the Q and tibiofemoral angles increase with hallux valgus [12]. However, these previous studies were conducted on older people, and it is possible that the alignment of the lower leg due to knee joint disease has an effect on hallux valgus. Furthermore, the relationship between hallux valgus and varus/valgus of the hindfoot is unclear.

Hypermobility of the tarsometatarsal (TMT) joint has been reported to be a pathological condition of hallux valgus [13, 14]. However, it is unclear whether hallux valgus causes hypermobility of the TMT joint or vice versa. The foot consists of many joints, including the TMT joint, and performs three-dimensional (3D) motion (dorsiflexion-plantarflexion, abduction-adduction, and eversion-inversion) and compound motion (pronation-supination). Feet with hallux valgus may differ, not only in TMT joint mobility, but also in the midfoot and hindfoot morphology compared to those without.

This study aimed to clarify the relationship between the morphology and mobility of the hindfoot with hallux valgus and explore the characteristics of the entire foot in young women with no injuries to the lower extremities.

## Methods

### Participants

This cross-sectional study was approved by the Ethics Committee of Fukushima Medical University (approval number: 2021–028) and involved female university students between 18 and 21 years of age. When comparing the two groups (those with and without hallux valgus) for hindfoot morphology and joint mobility, it was calculated that 64 participants per group were required, assuming a significance level of 0.05, power of 0.8, and effect size of 0.5. Since 29.7–41.5% of young women have hallux valgus [2, 3], it was necessary to measure 154–215 women to obtain 64 women with hallux valgus. Exclusion criteria were current outpatient treatment for lower extremity orthopedic disease and a history of knee and ankle surgery. The purpose of the study was explained to the participants both verbally and in writing, and informed consent was obtained.

### Measurement

#### Foot morphology

**Fig. 1:**
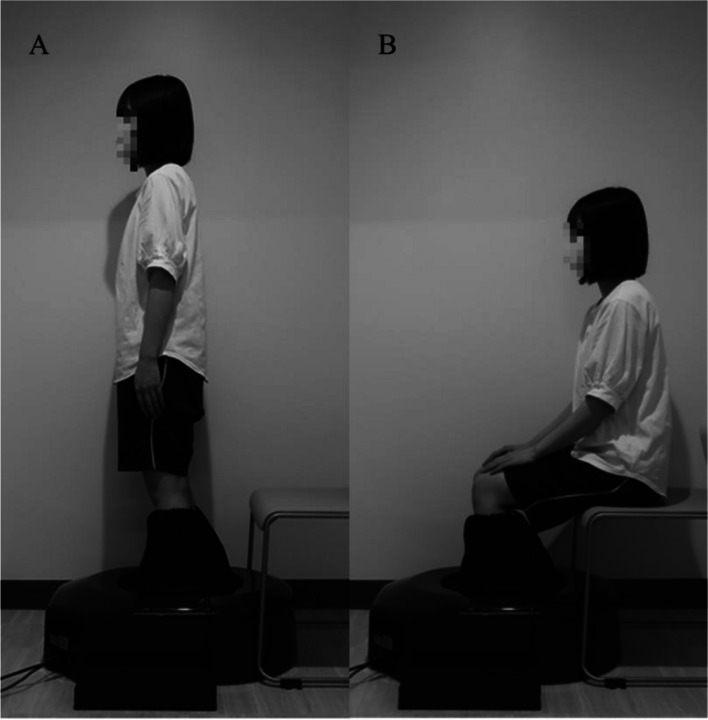
Measurement of foot morphology using foot-scanning system. **A** standing position, **B** sitting position

All measurements were performed barefoot. Foot morphology was measured using a simple, non-invasive 3D automatic footprint measurement apparatus (JMS-3100; Dream GP Inc., Osaka, Japan) (Fig. 1). This foot-scanning system uses a rail-type laser around the entire circumference of one foot to measure approximately 30,000 points on the foot, such as the instep, heel, toe, and sole. First, in a standing position, markers with a diameter of 5 mm were attached to the navicular tuberosity, bottom of the calcaneal tuberosity, and enthesis of the Achilles tendon [11]. Participants placed one foot in the measuring instrument, both feet shoulder-width apart, and stood still with their weight evenly distributed over both feet, and their foot shape was measured twice on each side. Next, the participants were seated in a chair, the hip joints were in adduction/abduction with internal/external rotation in a neutral position, and the knee and ankle joints were at 90°. Footprints were measured twice on each side. The first measurement value was adopted, but if the foot was moved during the measurement and the measurement could not be performed, the second measurement value was adopted.

**Fig. 2:**
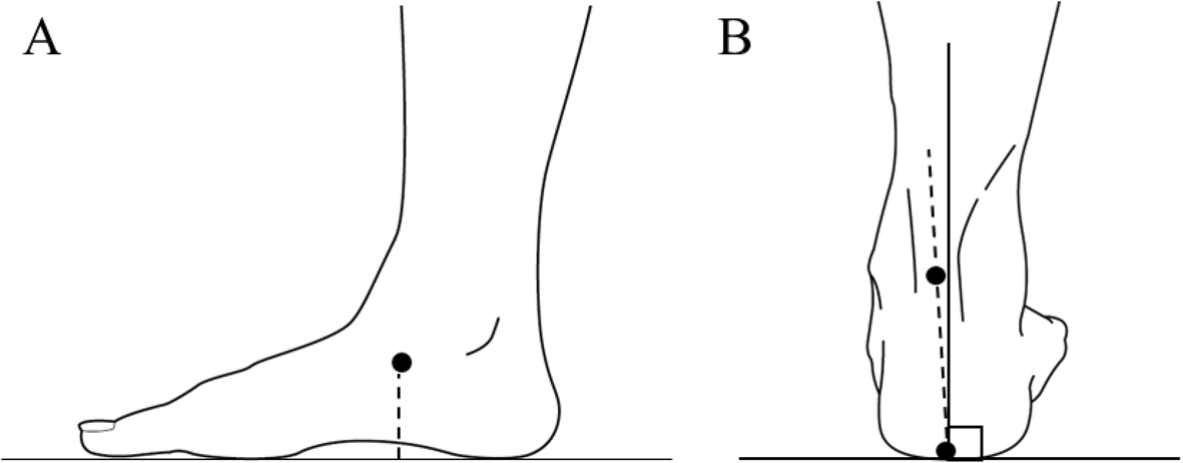
Parameters of foot morphology. **A** arch - height ratio, **B** heel - floor angle

The following parameters were measured for foot morphology. The hallux valgus angle was measured as the angle between a line connecting the head of the first metatarsal and the head of the first proximal phalanx and a line connecting the head of the first metatarsal and the posterior medial malleolus. The arch-height ratio was calculated as the ratio of the height of the navicular tuberosity from the floor to foot length (Fig. 2A). It has been reported that the navicular height of the body landmark divided by the foot length is highly correlated with radiographic measurements [15]. Heel-floor angle (HFA) was measured as the angle between the line connecting the bottom of the calcaneal tuberosity and enthesis of the Achilles tendon and the vertical line from the floor surface (Fig. 2B). The line connecting the bottom of the calcaneal tuberosity and enthesis of the Achilles tendon was measured as positive (+) when the line sloped inward from the vertical line from the floor and the sole turned outward (eversion) and negative (−) when the line sloped outward and the sole turned inward (inversion) [8]. In addition, the arch-height ratio and HFA were examined for differences between the values measured in a chair-sitting position and those measured in a standing position.

Change in arch-height ratio = arch-height ratio in a sitting position - arch-height ratio in a standing position.

Change in HFA = HFA in a sitting position - HFA in a standing position.

#### Ankle range of motion

**Fig. 3:**
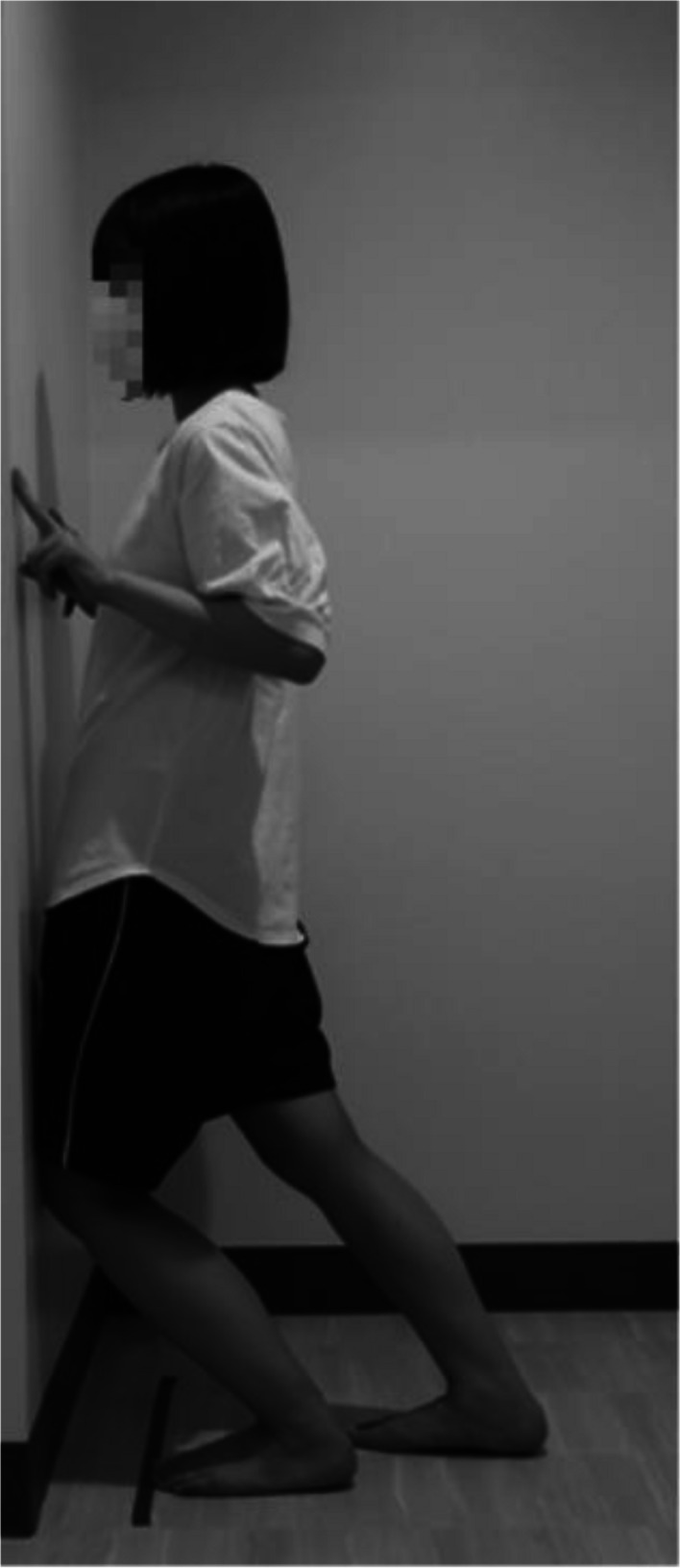
Ankle dorsiflexion range of motion

All ankle joint angles were measured barefoot by a female physical therapist. In this study, ankle dorsiflexion and plantarflexion range of motion (ROM) were measured using a method reported to be more reliable than using a goniometer [16, 17]. The ROM of ankle dorsiflexion was measured using the following method (Fig. 3). First, the participant stood facing the wall, such that their hallux was 10 cm away from the wall. Next, the lower leg was loaded on the side to be measured, the ankle joint was dorsiflexed without lifting the heel from the ground, and the knee joint was flexed, followed by confirmation as to whether the front of the knee could contact the wall. If this position was possible, the foot was moved from the wall in 1-cm increments to identify a position in which the knee touched the wall without lifting the heel. Conversely, if this posture was not possible, the foot position was moved closer to the wall in 1-cm increments. At this time, the position of the lower limbs that were not measured was not specified. The maximum distance from the wall to the tip of the hallux was measured twice as the dorsiflexion range of the ankle joint. The within-session intra-rater reliability (ICC_2,3_) estimate for the dorsiflexion range of the ankle joint has been reported to be 0.98–0.99 [16].

**Fig. 4:**
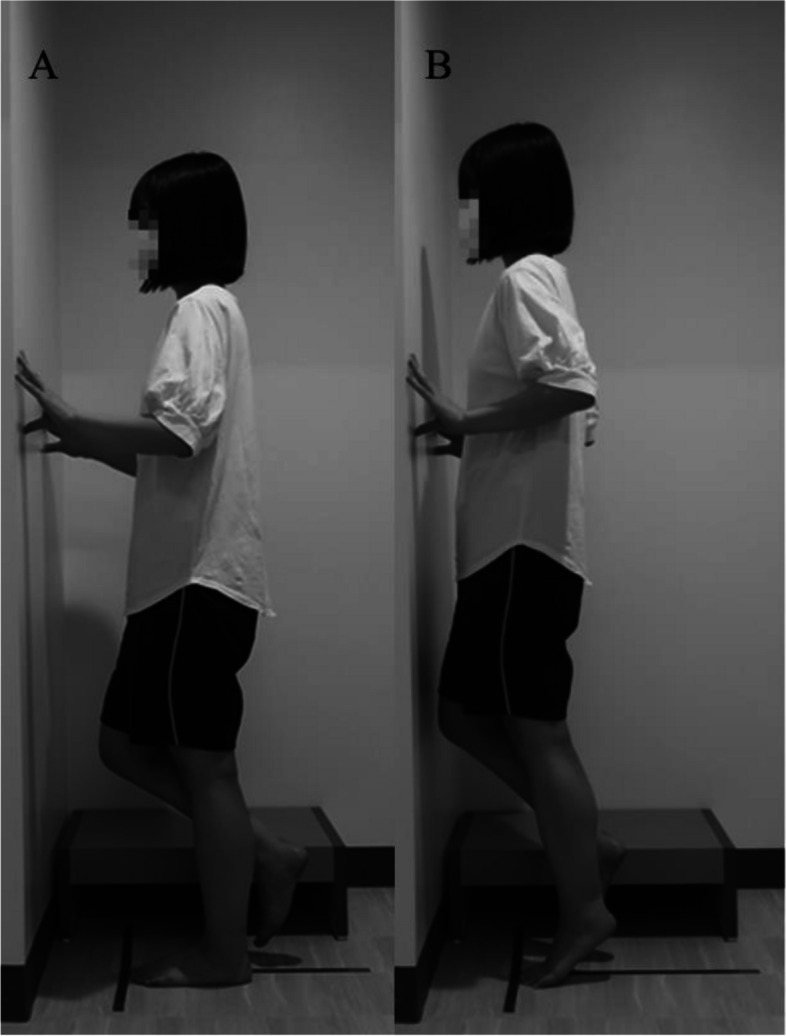
Ankle plantarflexion range of motion. **A** starting posture, **B** posture with the heel raised to the maximum

The ROM of the ankle plantarflexion was measured using the functional heel-rise test [17]. The participants stood facing the wall, with the tip of the hallux of both feet 15 cm away from the wall (Fig. 4A). Using a right-angle ruler, the position where the wall and the top of the head formed a right angle (90°) was specified, and the distance from this point to the floor was measured. Next, the participant stood on only the foot being measured, and the heel of the measured foot was maximally raised from the floor (Fig. 4B). The elbow joints on both sides were flexed at 90°, and the participants touched the wall with their fingertips of both hands to maintain balance. In this posture, the distance from the floor to the top of the head was measured. The difference between the maximum and starting height was calculated. This measurement was conducted on the left and right sides. It has been reported that for the plantarflexion range of the ankle joint, the within-session intra-rater reliability (ICC_2.1_) estimate was 0.99, and the inter-rater reliability (ICC_2.k_) was 0.79–0.87 [17].

**Fig. 5:**
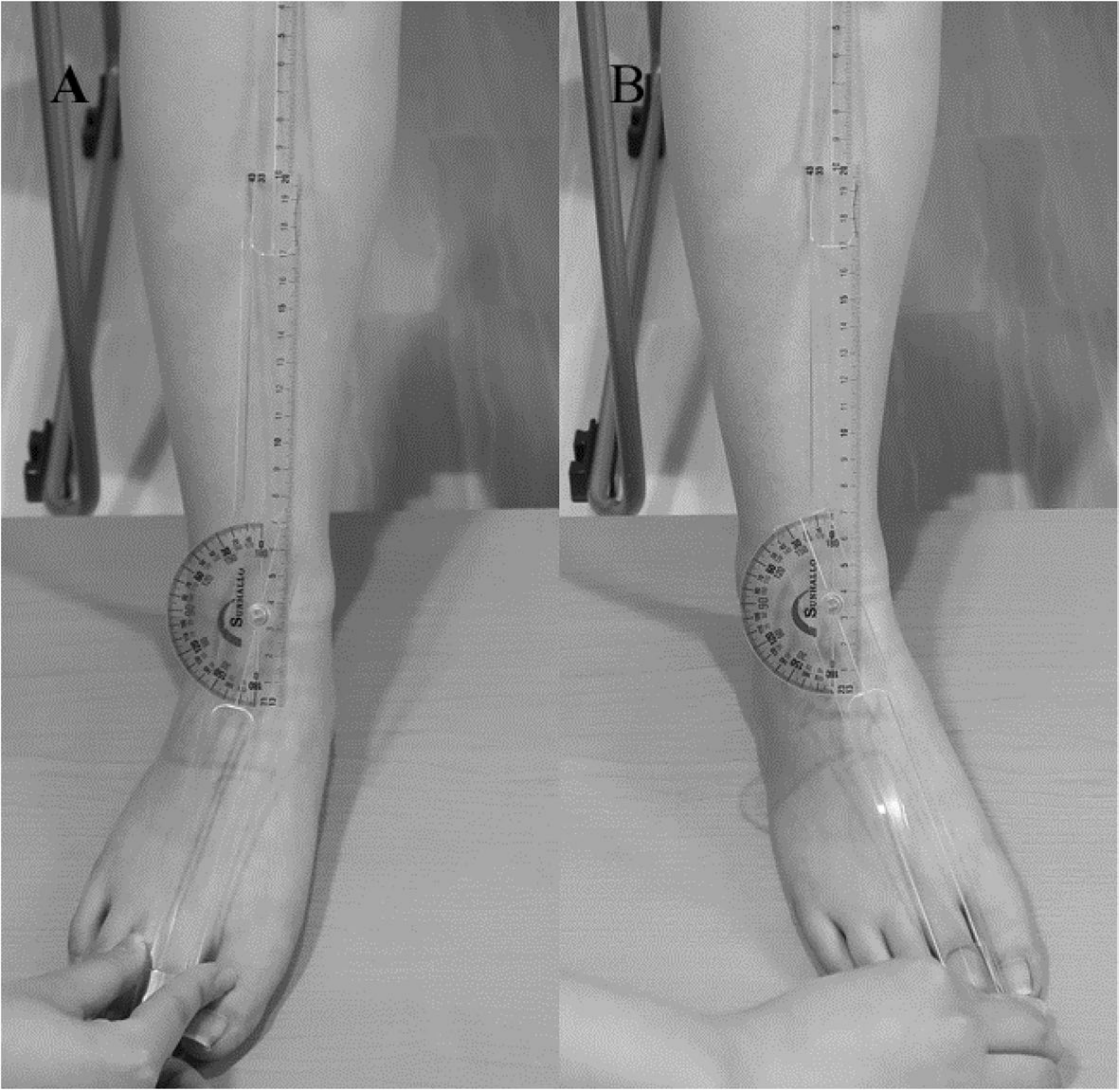
Ankle inversion and eversion range of motion. **A** eversion, **B** inversion

The angle of inversion and eversion of the ankle joint was measured using the following method (Fig. 5). The participants sat on a chair, and the ankle joint was in a comfortable relaxed position, usually in some plantarflexion, and moved passively. In the center of the medial and lateral malleolus, the angle of the crest of the tibia and the longitudinal midline on the anterior surface of the second metatarsal were measured using a goniometer. The left and right inward and outward inversions were measured twice. It has been reported that for the angle of inversion and eversion of the ankle joint, the within session intra-observer reliability (ICC_2.1_) ranged 0.82–0.96 and inter-observer reliability ranged 0.62–0.73 [18]. The ROM of all ankle joints was statistically processed using the maximum value.

### Statistical analysis

Participants were classified into two groups according to the presence of hallux valgus. According to the Japanese Orthopedic Association Clinical Practice Guidelines on the management of hallux valgus, a hallux valgus angle of ≥ 20° based on radiographic measurement values is judged to have hallux valgus [19]. A footprint-based hallux valgus angle of 16° has been reported to be equivalent to a radiograph-derived hallux valgus angle of 20° [20]. A hallux valgus angle of ≥ 16° on at least one side was judged to have hallux valgus. For statistical analysis, the measured values of either the left or the right foot were used [21]. In cases of bilateral or unilateral hallux valgus, the foot with the larger hallux valgus angle was selected. In cases where the angle of hallux valgus was < 16° bilaterally, a random number table was used to select the same number of left and right feet. We compared the arch-height ratio and HFA in the standing position, the amount of change in the arch-height ratio and HFA from the standing to the sitting position, and ROM of the ankle joint between the two groups with and without hallux valgus. Data normality was analyzed using Shapiro–Wilk test, and data were compared between groups using t-test or Mann–Whitney U test. Spearman’s correlation coefficient was used to analyze the relationship between foot morphology and ROM of the ankle joint in the groups with and without hallux valgus. Statistical analyses were performed using IBM SPSS for Windows (version 28.0; IBM Corp., Armonk, NY, USA), and the significance level was set at *p* < 0.05.

## Results

A total of 147 participants agreed to participate in this study. Of these, seven women with a history of knee and ankle surgery were excluded. Thirty-two women had bilateral or unilateral hallux valgus, and 108 had no hallux valgus. Table 1 presents the characteristics of the participants.

**Table 1:** Characteristics of participants

	No-HV *n* = 108		HV *n* = 32		*p*-value
Age (years)	19	(18–19)	19	(18–19)	0.144
Height (cm)	158.0	(154.0–161.0)	157.8	(152.9–161.2)	0.584
Body weight (kg)	51.2	(47.0–56.0)	48.8	(46.4–52.0)	0.027
BMI (kg/m^2^)	20.5	(18.9–22.5)	19.9	(18.7–21.1)	0.546

Median (interquartile range)

*HV* Hallux valgus, *BMI* Body mass index

Table 2 shows the foot morphology and ankle joint ROM depending on the presence of hallux valgus. The HFA was 3.44 ± 3.15° in participants without hallux valgus and 5.14 ± 3.48° in those with hallux valgus. The amount of change in arch-height ratio was 2.35 ± 1.02 mm/mm*100 in participants without hallux valgus and 2.86 ± 1.25 mm/mm*100 in those with hallux valgus. The degree of change in the arch-height ratio due to posture was significantly greater in participants with hallux valgus (*p* = 0.021). There was no significant difference in the ROM of the ankle joint between participants with or without hallux valgus.

**Table 2:** Comparison of foot morphology and ankle range of motion on the presence of hallux valgus

	No-HV *n* = 108	HV *n* = 32	*p*-value
Foot morphology			
HV angle	10.3 (7.9–13.3)	17.6 (16.8–19.9)	< 0.001
Arch-height ratio	17.61 ± 2.20	16.76 ± 2.63	0.069
HFA	3.44 ± 3.15	5.14 ± 3.48	0.010
Change in arch-height ratio	2.35 ± 1.02	2.86 ± 1.25	0.021
Change in HFA	-2.15 ± 3.14	-3.35 ± 2.87	0.055
Ankle joint ROM			
Dorsiflexion	14.3(13.0–16.2)	15.3(13.4–18.1)	0.199
Plantarflexion	7.7(6.7–8.5)	7.5(6.8–8.5)	0.546
Inversion	42.5(38.0–53.5)	50.0(42.0–55.0)	0.133
Eversion	24.0(20.0–30.0)	23.5(20.0–32.0)	0.913

Unit: HV angle, HFA, change in HFA, inversion, and eversion: degree; arch-height ratio and change in arch-height ratio: mm/mm*100; dorsiflexion and plantarflexion: cm

Median (interquartile range) or mean ± standard deviation

*HV* Hallux valgus, *HFA* Heel-floor angle, *ROM* Range of motion

Table 3 shows the correlation between foot morphology and ankle joint ROM depending on the presence of hallux valgus. In the group without hallux valgus, no relationship was observed between foot morphology and ankle ROM. In the hallux valgus group, the change in arch-height ratio and HFA was correlated with the ROM of eversion (*r* = 0.391, *p* = 0.027; *r* = -0.362, *p* = 0.042).

**Table 3:** Relationship between foot morphology and ankle joint range of motion depending on the presence of hallux valgus

No-HV *n* = 108				
	Dorsiflexion	Plantarflexion	Inversion	Eversion
HV angle	0.089	0.022	-0.191^*^	0.135
Arch-height ratio	0.010	0.118	-0.124	-0.009
HFA	0.115	-0.065	0.055	-0.096
Change in arch-height ratio	-0.112	-0.126	0.138	0.108
Change in HFA	-0.172	0.221^*^	-0.174	-0.006
HV *n* = 32				
	Dorsiflexion	Plantarflexion	Inversion	Eversion
HV angle	0.195	-0.009	0.031	-0.013
Arch-height ratio	-0.126	-0.250	-0.156	-0.089
HFA	0.026	0.210	0.210	0.082
Change in arch-height ratio	-0.032	-0.002	0.308	0.391^*^
Change in HFA	0.027	0.275	-0.112	-0.362^*^

*HV* Hallux valgus, *HFA* Heel-floor angle

*p < 0.05

## Discussion

We compared the hindfoot morphology and ankle joint of ROM of young women with and without hallux valgus. The HFA in participants with hallux valgus was tilted inward, and the arch-height ratio decreased significantly when the sitting position was changed to the standing position. There was no difference in the ROM of the ankle joint with or without hallux valgus; however, with hallux valgus, there was a relationship between the angle of eversion on the frontal plane and the amount of change in arch-height ratio and HFA.

Regarding the fact that the calcaneus tilts inwards in participants with hallux valgus, radiographic observation of the hindfoot alignment of hallux valgus reveals valgus deviation of the subtalar joint [22]. Furthermore, as observed using 3D computed tomography, large hallux valgus angles result in hindfoot bones that easily rotate in the everting direction. As a result, it has been reported that the talus may induce greater internal rotation of the first metatarsal [23]. Hindfoot valgus is associated with pronation of the first metatarsal, and conversely, hindfoot varus is associated with supination of the first metatarsal, assessed using weight-bearing computed tomography [24]. The morphology of the hindfoot is considered to be affected by the knee joint and middle foot. The participants in this study were young women who were not outpatients for lower extremity orthopedic disease at the time of the study and had not received knee or ankle surgery in the past. Among those with no knee joint problems, the findings confirmed that the foot with hallux valgus had a larger HFA, that is, the calcaneus tilted inward.

In addition, those with hallux valgus had a significantly greater amount of change due to posture with arch-high ratio than those without hallux valgus. In a study of healthy young people, it was reported that the arch-height index and flexibility changed depending on the weight-bearing load [25], and it was confirmed that the arch changes with the weight-bearing load. Hypermobility of the first ray, which consists of the medial cuneiform bone, first metatarsal bone, and hallux – forming the arch – has been reported as a related factor in hallux valgus, although the causal relationship is unclear [13]. According to computed tomography imaging of hallux valgus in a standing position, pronation of the first metatarsal bone is more affected than that of the first TMT joint [5]. Therefore, in the present study, since the height of the navicular bone was measured as the arch height, the part of the bone that constitutes the medial arch was measured. Considering the chain reaction of the bones that form the arch, the large change in arch-height ratio in the hallux valgus group suggests that the bones that form the arch are highly mobile, as in other reports.

There was no difference in the ROM of the ankle joints with or without hallux valgus. A correlation has been reported between significantly increased ROM of the hip joint internal rotation, ankle dorsiflexion/plantarflexion, and first metacarpophalangeal joint dorsiflexion, together with significantly increased Q, tibiofemoral, and rearfoot angles, and the presence of hallux valgus [12]. However, the above studies were limited by their small sample size, wide age range of the participants, and use of clinic attendees as the control group. In comparison, the participants of the current study were of a young age, and there seems to be no effect of age-related osteoarthritis. In addition, in this study, the ROM of the ankle dorsiflexion [16] and plantarflexion [17] was measured using a method that has been confirmed to be more reliable than or equal to angle measurement using a goniometer. Therefore, the current results may have differed from those of previous reports because of the use of different measurement tools. On the other hand, at a hallux valgus angle of ≥ 16°, the amount of change in arch-height ratio and HFA correlated with the ROM of eversion. It is presumed that hypermobility of the navicular and calcaneus bones that make up the medial arch increases ROM of eversion of the foot.

This study has several limitations. First, the footprint measurement was not based on radiographs because it was performed in healthy women. Therefore, it may be difficult to compare all results with radiographs because an index that can be perceived from the body surface was used. Second, the ROM of the ankle joint was measured using a non-invasive method. The ankle is a complex composed of the ankle joint and the subtalar joint [26], and the movement of the ankle joint consists of multi-joint compound movements. In the assessment of ROM of the ankle joint, it is impossible to separate the movements of each joint, including the subtalar joint. Third, inversion and eversion ankle range of motion are movements of the frontal plane. However, the highly reliable measurement method with the goniometer is where the ankle was relaxed in comfortable plantarflexion. Therefore, it may be slightly different from the motion of the frontal plane. Fourth, the amount of weight-bearing load on the sole was not specified in the measurement of the arch-height ratio and HFA in the sitting position. It is not possible to compare the change in weight-bearing load from sitting to standing with the changes in arch height and HFA. Fifth, because arch height is a measure of navicular tubercle height, it may be difficult to compare with studies that directly assessed first-ray mobility. Sixth, 140 women were available for analysis, and while the sample size varied according to incidence, it was slightly under-represented in this study.

## Conclusions

The results of this study indicated that, in feet with hallux valgus, HFA tilts inward, and the amount of change in the arch-height ratio increases depending on the posture. The amount of change in arch-height ratio and HFA correlate with the eversion angle, suggesting hypermobility of the bone joints that form the medial arch. To prevent hallux valgus from a young age, it is considered desirable to appropriately utilize footwear and insoles that support the arch and simultaneously suppress the inclination of the calcaneus.

## Data Availability

The datasets generated and analyzed during the current study are not publicly available because we did not obtain consent from the participants to publish the individual data. The datasets are available from the corresponding author upon reasonable request.

## Abbreviations

TMT joint: Tarsometatarsal joint
3D: Three-dimensional
HFA: Heel-floor angle
ROM: Range of motion
